# Toxicity of the Anti-ribosomal Lectin Ebulin f in Lungs and Intestines in Elderly Mice

**DOI:** 10.3390/toxins7020367

**Published:** 2015-02-02

**Authors:** Manuel Garrosa, Pilar Jiménez, Jesús Tejero, Patricia Cabrero, Damián Cordoba-Diaz, Emiliano J. Quinto, Manuel J. Gayoso, Tomás Girbés

**Affiliations:** 1 Cell Biology, Histology and Pharmacology, Faculty of Medicine and INCYL (Institute of Neurosciences of Castile and Leon), University of Valladolid, Valladolid E-47005, Spain; E-Mails: garrosa@med.uva.es (M.G.); gayoso@med.uva.es (M.J.G.); 2 Nutrition and Food Science, Faculty of Medicine, University of Valladolid, Valladolid E-47005, Spain; E-Mails: pilarj@bio.uva.es (P.J.); jesus.tejero@uva.es (J.T.); patricia_cabrero@hotmail.com (P.C.); equinto@ped.uva.es (E.J.Q.); 3 CINAD (Center for Research in Nutrition, Food and Dietetics), University of Valladolid, Valladolid E-47005, Spain; 4 Pharmacy and Pharmaceutical Technology, Faculty of Pharmacy and IUFI (Institute of Industrial Pharmacy), Complutense University of Madrid, Madrid E-28040, Spain; E-Mail: damianco@farm.ucm.es

**Keywords:** ebulin f, *Sambucus ebulus*, ribosome-inactivating proteins, elderly mice, toxicity

## Abstract

All parts of dwarf elder (*Sambucus ebulus* L.) studied so far contain a ribosome-inactivating protein with lectin activity (ribosome-inactivating lectin; RIL), known as ebulin. Green fruits contain ebulin f, the toxicity of which has been studied in six-week-old mice, where it was found that the intestines were primary targets for it when administered intraperitoneally (i.p.). We performed experiments to assess whether ebulin f administration to six- and 12-month-old mice would trigger higher toxicity than that displayed in six-week-old mice. In the present report, we present evidence indicating that the toxicological effects of ebulin f after its i.p. administration to elderly mice are exerted on the lungs and intestines by an increased rate of apoptosis. We hypothesize that the ebulin f apoptosis-promoting action together with the age-dependent high rate of apoptosis result in an increase in the lectin’s toxicity, leading to a higher lethality level.

## 1. Introduction

Aging is a multi-factorial process involving changes in the structure and function of organs and tissues; among these, vital organs, such as the lungs and intestines, are of particular interest. The small intestine of mammals has very important functions, such as the digestion of food and the absorption of the resulting nutrients [1,2,3]. The main functions of the colon are the absorption of water and electrolytes and to allow the fermentation of undigested macronutrients by the intestinal microbiota. From a histological point of view, the small intestine has a highly specialized and efficient tissue structure that has a very large surface made up of intestinal folds, villi, microvilli and crypts. Epithelial cells in the gastrointestinal tract have a high turnover rate, and the maintenance of normal cellular balance through apoptosis is crucial for the preservation of normal cellular function [4]. It has been reported that the aged small intestine displays hyper proliferation and a high rate of apoptosis to prevent structurally and functionally stable alterations [5].

Structural changes and cell turnover in the epithelial lining of the small intestine are regulated by apoptosis, which contributes to maintaining gut function [5,6]. The surface area of the small intestine decreases due to the degeneration of villi [7]. Associated with this are changes in the absorption of nutrients, such us glucose, vitamins, calcium, magnesium, zinc and copper [7,8,9]. This should be a determining factor in drug absorption for low permeable drugs in the different regions of the gastrointestinal tract. Despite the importance of the gut for health maintenance, the molecular and cellular mechanisms involved in the apoptotic process have not been completely explained.

Ebulins are anti-ribosomal lectins obtained from different parts of dwarf elder (*Sambucus ebulus* L.) [10,11]. Dwarf elder has been used since ancient times, and it is known to have pharmacological properties, namely antioxidant, anti-inflammatory, anti-rheumatic, anti-hemorrhoid and anti-infectious activities, among others [12,13,14,15]. A few of the molecules responsible for these activities have been identified, but at the same time, different components of the plant may be toxic if consumed in excess, which is probably due to the presence of ebulins [16,17]. We have previously reported that oral ingestion of ebulin f at 5 mg/kg of body weight killed 50% of the animals in 10 days, while those that survived recovered after 30 days from the administration of the toxin [10]. We obtained similar results following nasal administration of ebulin blo at the same dose [18]. We estimated, therefore, an approximate LD_50_ of 5 mg/kg of body weight for both oral and nasal administration, in contrast to the LD_50_ value of 2.8 mg/kg body weight for i.p. administration [11].

Ebulin f, present in dwarf elder fruit, is a type-2 ribosome-inactivating protein (RIP), *i.e.*, it is composed of two polypeptide chains, one a catalytic A type with *N*-glycosidase activity (E.C.3.2.2.22), and a B chain, linked by a disulfide bond, with sugar-binding capacity; this confers the nature of lectin on the whole RIP [19,20,21]. For this reason, these proteins are also called ribosome-inactivating lectins (RILs). It has been proposed that the RIP in the plant plays a protective role against insects, viruses and fungi [19]. Regarding the molecular action mechanism, ebulins cause the irreversible inactivation of ribosome by arresting its 28S rRNA, while its B-chain selectively bonds to d-galactose [19,22]. Ebulins are structural and functionally related to ricin [19,22]. The latter is one thousand-times more cytotoxic than ebulin in mice due to the great ability of its B chain to bind to the polysaccharide chains situated on the surface of plasma membrane proteins [22,23].

Previous studies have described the toxicity of ebulin f and ebulin blo in six-week-old mice [11,18]. Early intestinal targets were the transit amplifying cells present in the small intestine crypts, as well as the cells of the large intestine crypts. In both cases, the cellular action mechanism was the promotion of apoptosis [11]. The presence of these proteins in certain food products, the concern regarding their possible criminal use or as a bioweapon and also their potential application in medicine make the toxicology of these proteins an area of great interest [18].

It is well known that the organic characteristics of living beings vary with age, and in toxicology, in particular, the effects occurring throughout the different stages of life are noteworthy. As pointed out above, an increase in apoptosis is associated with aging, and we hypothesized that ebulin f would increase that process even more and that, therefore, it would increase its toxicity.

Ebulin f was isolated by affinity chromatography and characterized by mass spectrometry, as reported previously [10,24]. Its toxicity was first studied in six-week-old mice, and it was found that the intestines were the primary targets for ebulin f when administered by i.p. [11]. Since the small intestine of aged mice was reported to undergo a higher rate of apoptosis, we performed experiments to assess whether ebulin f administration to six- and 12-month-old mice would trigger higher toxicity than that displayed in six-week-old animals. In this paper, we report the toxicological effects of ebulin f in elderly animals after its administration via the intraperitoneal (i.p.) route, showing the toxicity exerted on the lungs and intestines and discussing the differences *vis-à-vis* young mice.

## 2. Results

**Figure 1:**
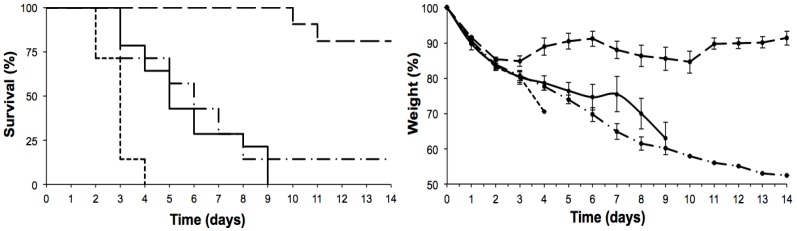
Effects of the intraperitoneal administration of ebulin f in six-week-old Swiss mice on the evolution of their survival (Kaplan-Meyer plots) (**left**) and body weight (**right**). Seven six-week-old mice per group were injected with ebulin f at 5.0 (dotted line), 3.1 (continuous line), 2.8 (dash-point line) and 2.5 mg/kg (dashed line) of body weight.

As already described, and as shown in Figure 1, six-week-old mice showed sensitivity to the intraperitoneal administration of ebulin f. An interesting effect observed in all of the experiments concerns the reduction in body weight promoted by the toxin. Two weight reductions were observed in the surviving animals; that is, after the first and the sixth days of treatment. The recovery period in both cases was 3–4 days.

**Figure 2:**
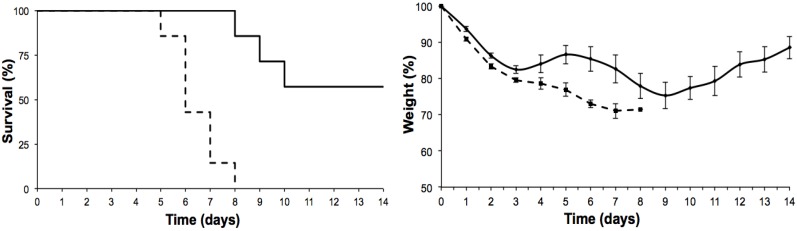
Effects of intraperitoneal administration of ebulin f in six-month-old Swiss mice on the evolution of their survival (Kaplan-Meyer plots) (**left**) and body weight (**right**). Seven six-month-old mice per group were injected with ebulin f at 2.1 (dashed line) and 1.4 mg/kg (continuous line) of body weight.

Six-month-old mice appeared to be more sensitive to ebulin f administration than six-week-old mice. As shown in Figure 2, the injection of ebulin f at 2.1 mg/kg of body weight killed all of the animals in eight days. After 14 days, the same concentration neither killed the mice nor produced signs of apparent tissue damage, as previously reported [11]. The administration of ebulin f at 1.4 mg/kg of body weight also triggered toxicity, leading to the death, after 14 days, of almost half of the six-month-old treated mice.

**Figure 3:**
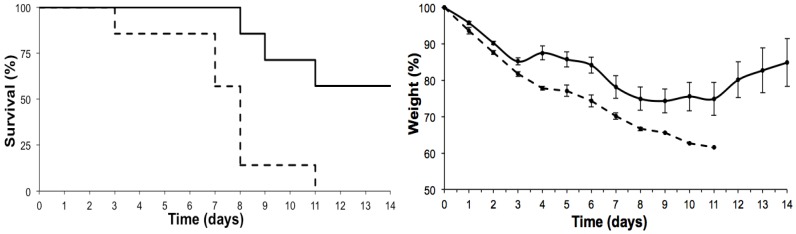
Effects of intraperitoneal administration of ebulin f in 12-month-old Swiss mice on the evolution of their survival (Kaplan-Meyer plots) (**left**) and body weight (**right**). Seven 12-month-old mice per group were injected with ebulin f at 2.1 (dashed line) or 1.4 mg/kg (continuous line) of body weight.

The population of 12-month-old mice revealed a sensitivity to ebulin f, which was very similar to that displayed by the six-month-old mice (Figure 3).

In order to ascertain the target(s) and the nature of the damage inflicted by the toxin, we performed a histological analysis of the intestines and lungs, depending on the lesions observed in young mice, of ebulin f-treated and untreated six-month-old and 12-month-old mice.

### 2.1. Effects on the Lung

**Figure 4:**
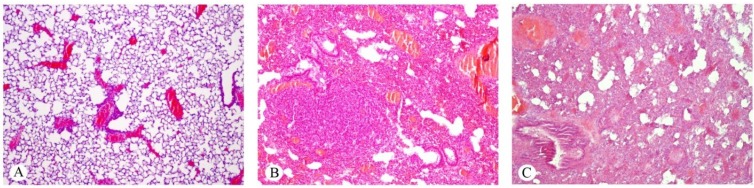
Histological sections of lungs from control (**A**) and experimental mice injected with 1.4 mg/kg of ebulin f at the age of six months (**B**) and 12 months (**C**). Venous congestion can be observed in the three groups, but whereas the structure of the lung appears normal in the control specimens, pneumonia in an advanced phase of hepatization is present in (**B**) and (**C**). Hematoxylin and eosin. Magnification ×25.

**Figure 5:**
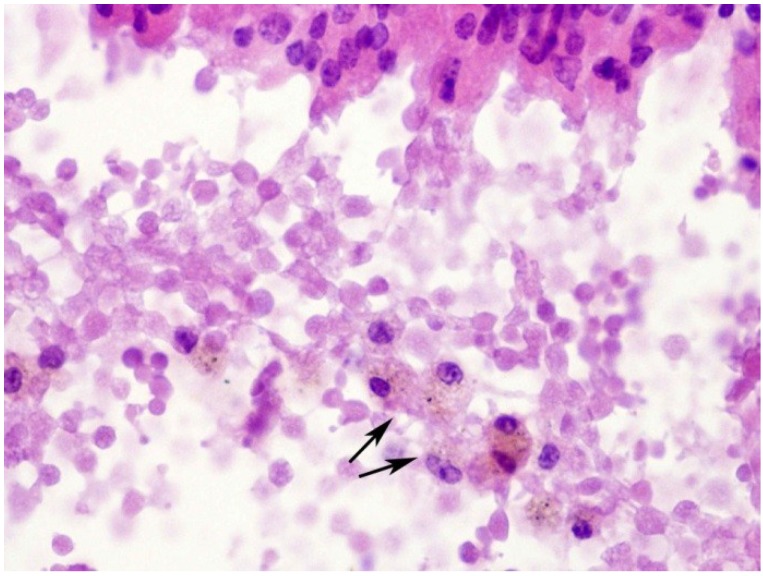
Histological section of the lung from a 12-month-old mouse injected with 2.1 mg/kg of ebulin f. Hemorrhage is present inside a bronchus lumen in a lung with pneumonia in the red hepatization phase. Erythrocytes have lost their eosinophilia and are being phagocytized by macrophages (arrows). Hematoxylin and eosin. Magnification ×370.

**Figure 6:**
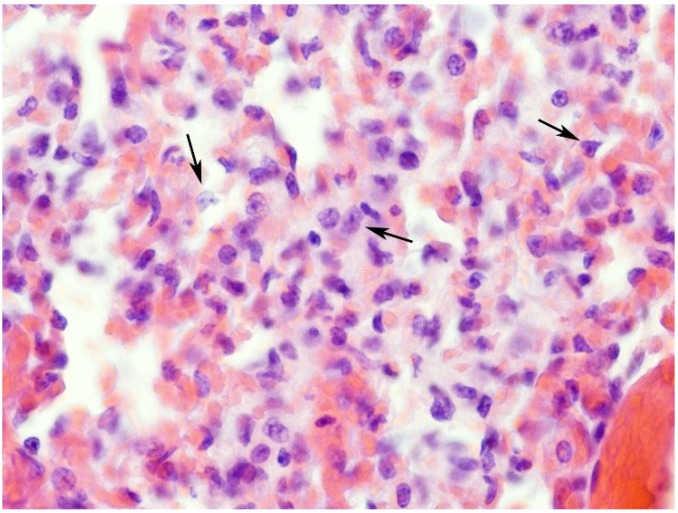
Histological section of a lung from a 12-month-old mouse injected with 1.4 mg/kg of ebulin f. Venous and capillary congestion can be seen in a pneumonic focus showing a chronic inflammatory infiltrate. Atypical nuclei with dysplasia (arrows) can also be observed. Hematoxylin and eosin. Magnification ×400.

Venous and capillary congestion was a common finding in both the control and experimental animals, but to a greater extent in the latter (Figure 4). In a few experimental specimens, pneumonia appeared in its initial phase, while others displayed hemorrhage during red hepatization (Figure 5), whilst in some animal, lungs appeared in the grey hepatization phase. The condensation areas, also including a chronic inflammatory infiltrate and thickening of the alveolus-capillary wall, seemed to be independent of the dose of toxin injected, but were more severe in 12-month-old animals, with the appearance of certain atypical nuclei with dysplasia (Figure 6).

### 2.2. Effects on the Intestines

**Figure 7:**
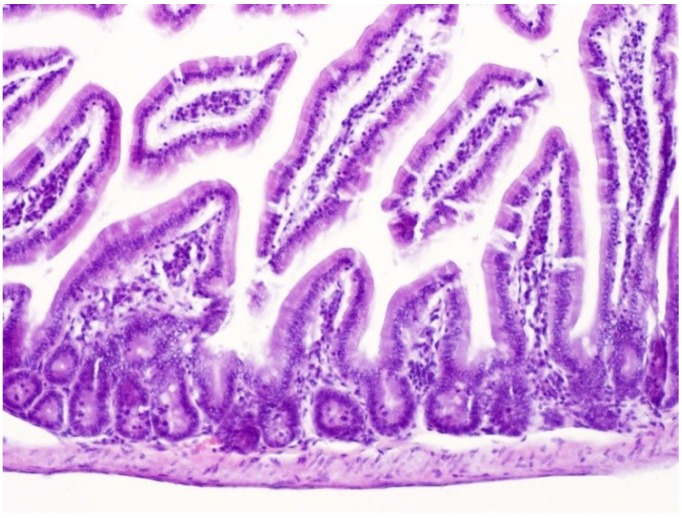
Histological section of the small intestine in a 12-month-old control mouse. An enlarged villus can be perceived. Hematoxylin and eosin. Magnification ×100.

**Figure 8:**
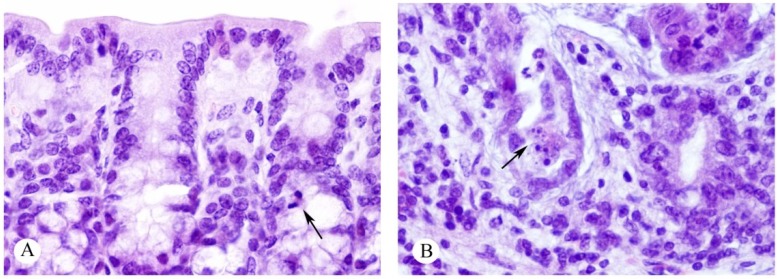
Histological sections of large intestine in 12-month-old mice in the control group (**A**); and injected with 2.1 mg/kg of ebulin f (**B**). Normal Lieberkühn’s crypts with mitoses (arrow) can be seen in A; crypts appear atrophic and show apoptosis (arrow) in (**B**). Hematoxylin and eosin. Magnification ×285.

In both control and experimental animals injected with 1.4 mg/kg of ebulin f, the histological structure of the small and large intestines appeared normal, except for some villi showing a considerable enlargement in width (Figure 7). Experimental animals injected with doses equal to or higher than 2.1 mg/kg underwent a mild atrophy of Lieberkühn’s crypts, displaying apoptosis and the loss of enterocytes (Figure 8).

The decrease in cell renewal and resulting atrophy lead to a shortening of both intestines. The small intestine in the animals injected at six months of age was observed to have shortened about 4 cm (*p* < 0.05) when compared with control animals of the same age; meanwhile, the length of the large intestine in animals injected at 12 months of age decreased approximately 2.3 cm (*p* < 0.05) *vis-à-vis* their respective age-matched control animals (Table 1). In contrast, differences between small intestine lengths at 12 months old, as well as between large intestine lengths at six months old were not statistically significant in accordance with one-way ANOVA.

**Table 1:** Mean ± SD of the length (cm) of the small and large intestines in the different groups. (*) and (^#^) the statistical difference (*p* < 0.05) by means of one-way ANOVA.

Organ	Control 6 m	Control 12 m	6 m	12 m
Small intestine	32.20 ± 3.63 *	34.10 ± 1.20	28.00 ± 2.40 *	29.75 ± 1.70
Large intestine	9.42 ± 0.57	11.01 ± 0.70 ^#^	8.75 ± 0.84	8.75 ± 0.86 ^#^

## 3. Discussion

In previous studies, we reported that 2.5 mg/kg of ebulin f administered i.p. did not kill the young mice up to at least 14 days after injection, whilst 5.0 mg/kg killed all of the animals quickly (2–4 days) [11]. In this study, we present evidence that adult mice (six-month- and 12-month-old animals) are more sensitive to ebulin f administration than young mice (six-week-old animals). Our findings show that ebulin f administered i.p. to elderly mice causes dramatic damage to the lungs, with the development of pneumonia, and also affects the intestines, with loss of enterocytes and atrophy of Lieberkühn’s crypts. Digestive mucosal damage has been described after i.p. administration in six-week-old mice [11], as well as upon intravenous injection of related RILs, such as nigrin b and ricin [25,26,27,28,29]. The lowest dose used in this study (1.4 mg/kg) did not cause damage to the intestines, although it was enough to cause considerable damage to the lungs. Doses of 2.1 mg/kg and higher were seen to be quite toxic for all of the organs studied.

Hitherto, there has been no description of pneumonic foci after administration of ebulin f in young mice, in which even higher doses than those used by us (5.0 mg/kg) caused only a focal congestion in the lungs. To explain this differential effect, we must consider the lower resistance of the lungs to hazards in more aged animals, since a six-month-old mouse is already rather old, more or less comparable to a man in his sixties; besides, the lung damage found in our study was more severe in 12-month-old animals than in six-month-old ones. Contrastingly, regarding the intestines, no significant differences were found in the histological changes caused by ebulin when comparing six- *versus* 12-month-old mice.

We have also reported in this study the appearance of atypical nuclei showing dysplasia in lung parenchyma following administration of ebulin f (Figure 6), and although we have not observed proliferative changes, the presence of these atypical nuclei may raise concerns regarding a possible mutagenic effect of this lectin.

The toxicity of ebulin f as compared to ricin is considerably lower when injected i.p., and this difference could be attributed to the different intracellular route followed by both toxins. Ebulin f, in the same way as nigrin b, goes from endosomes to lysosomes, where it suffers a degradation that may transform it into inactive products ready to be expelled from the cell, whereas ricin seems to follow a retrograde transport from endosomes to the trans-Golgi network and a final transfer to the rough endoplasmic reticulum [30].

Concerning the action mechanism, the effects of ebulin f, similar to those of the related RIL nigrin b, do not seem to be of the type expected simply from an arrest of protein synthesis triggered by a typical translation inhibitor, since the concentration of these proteins required to inhibit translation is higher than that needed to affect cell viability [31,32,33]. Ebulin f promoted apoptosis in the intestines [11]. In addition, it has been reported that aging in mice promoted accelerated apoptosis, leading to the breakdown of intestinal mucosa [34]. This seems important to explain the apoptosis-dependent derangement promoted by ebulin f. In our case, we hypothesize that ebulin f apoptosis-promoting action together with the age-dependent high rate of apoptosis result in a higher toxicity level of the lectin, leading to a higher lethality level.

The effects exerted by ebulin f on mice reported here support the notion that they might be pleiotropic and probably comprise transfer arrest along with the promotion of other mechanisms that trigger apoptosis. Additionally, it has recently been reported that type-2 RIP cytotoxicity is mediated by the unfolded protein activated in response to endoplasmic reticulum stress [35]. Furthermore, ricin poisoning promoted the differential expression of at least six proteins in the lungs of mice exposed to aerosolized ricin [36].

Work is still underway to analyze in mice the behavioral pattern of cytokines, the activity of the caspase system following administration of sub-toxic and toxic concentrations of ebulin f and the potential changes in lung protein expression due to ebulin f toxicity. As a general consideration regarding the toxicity described and when taking into account the traditional ingestion of dwarf elder fruits, it becomes most important to employ methods to detoxify ebulin, such as boiling the aqueous extracts of the plant to a level safe enough to inactivate the protein and render it sensitive to degradation by acidic pepsin, as has recently been reported [37].

## 4. Experimental Section

### 4.1. Materials

All common chemicals and biochemical compounds were of the highest purity available and most were purchased from Sigma-Aldrich (Madrid, Spain). Isoflurane was purchased from Esteve Veterinaria (Barcelona, Spain). The chromatographic supports for protein isolation were purchased from GE Healthcare Europe GmbH (Barcelona, Spain). The affinity chromatographic support was acid-treated Sepharose 6B and was prepared as described elsewhere [10]. Dwarf elder (*Sambucus ebulus* L.) green fruits were harvested in Barruelo del Valle (Valladolid, Spain) during July and August and stored frozen at −20 °C until use. Green fruits were used for isolating ebulin f, as they contain the largest amount of that protein.

### 4.2. Isolation of Ebulin f from *S. ebulus* Fruits

Highly purified ebulin f was prepared essentially as previously described elsewhere [24] with a slight modification that is very important to preserve the flow properties and clearness of the chromatography columns [10]. Briefly, 200 g of frozen green fruits collected in July were minced and ground in a mortar to obtain a paste material, which was extracted overnight with 800 mL of extraction buffer (280 mM NaCl containing 5 mM sodium phosphate, pH 7.5). This pasty extract was strained through two layers of cheesecloth, and the fluid was centrifuged at 7500× *g* for 45 min at 4 °C. The supernatant was centrifuged a second time at the same speed for 30 min and subsequently removed and filtered through two layers of common laboratory filter paper to remove the mucilaginous material. This simple procedure ensures its removal without the quality and activity of the protein preparation being affected. The filtered extract was chromatographed through acid-treated Sepharose 6B (AT-Sepharose 6B) to obtain d-galactose-binding proteins, as previously reported for the lectins of dwarf elder fruits [10]. Seven hundred fifty milliliters of extract were applied to an XK50 (5 × 15 cm) column (GE-Pharmacia) containing 200 mL of freshly prepared AT-Sepharose 6B equilibrated with 280 mM NaCl and 5 mM Na-phosphate (pH 7.5) buffer. The column was washed with the same buffer until the A_280_ reached values close to 0, and the protein fraction not retained by the AT-Sepharose 6B column was discarded. The retained protein fraction was further eluted with the same buffer containing 0.2 M lactose. Fractions of 10 mL were collected, and those containing proteins were pooled and concentrated with an Amicon system using a Y10 membrane down to 5 mL. The concentrated protein was applied to a Superdex 75 (26/60; GE) column equilibrated with 400 mM NaCl and 5 mM Na-phosphate (pH 7.5) buffer and was eluted with the same buffer at 2.5 mL/min. The first 70 mL were discarded, and then, fractions of 2.5 mL were taken and their A_280_ measured. The fractions containing protein were pooled, dialyzed against water and finally concentrated as above to reach concentrations of 2.5–4.0 mg/mL. The purity of the ebulin f was assessed by SDS-PAGE and mass spectrometry, as indicated elsewhere [10].

### 4.3. Animals and Experimental Groups

Treatments, experiments and euthanizing the mice were conducted according to the European Communities Council guidelines [38] concerning the protection of laboratory animals. A total of 56 female Swiss mice were used: four experimental groups (*n* = 7) of 6-week-old, two experimental groups (*n* = 7) of 6-month-old and two experimental groups (*n* = 7) of 12-month-old animals. The number of animals per group was reduced to minimize unnecessary killing. Mice were obtained from our university facilities, housed individually in plastic cages in a temperature-controlled room and fed *ad libitum* with free access to water in a 12 light-dark cycle.

### 4.4. Treatment with Ebulin f

Water-dissolved ebulin f adjusted to the required concentrations for the mice’s weight was i.p. administered (60 µL) to Swiss mice under mild isoflurane anesthesia for easier handling. Control mice under the same experimental conditions, but with water, were not affected either with respect to survival or body weight. Six-month-old experimental animals were i.p. injected with a single dose of either 1.4, 2.1, 2.8 or 5.0 mg/kg ebulin f dissolved in 0.1 M phosphate buffer saline (PBS) pH 7.4, while 12-month-old animals were injected also with a single dose of either 1.4 or 2.1 mg/kg of ebulin f in the same conditions. Dead animals were immediately taken for histological study. Two weeks after administration of the toxin, all the surviving animals were anesthetized with isoflurane and sacrificed by decapitation. The dead animals were then counted to construct the corresponding Kaplan-Meyer plots.

### 4.5. Histological Analysis

The lungs and intestines were removed from the dead mice and fixed by immersion in 4% buffered paraformaldehyde. At least four animals were used for the histological analysis. Samples of the inferior lobe of the left lungs were taken, while the small and large intestines were measured for length and then sampled systematically 1 cm from the pylorus and ileocecal valve, respectively. Pieces were processed for paraffin embedding, and 7 µm-thick slices were obtained with a Minot microtome and stained for light microscopy with hematoxylin and eosin or with Masson’s trichrome. Photomicrographs were taken with a Spot digital camera in an Axiophot Zeiss photomicroscope.

### 4.6. Statistical Analysis

Numeric data were statistically analyzed by means of one-way ANOVA. The significance level was established at *p* < 0.05.
